# Clinical study on the effects of the applied volume of moisturizer in patients with asteatosis

**DOI:** 10.1111/1346-8138.16160

**Published:** 2021-09-21

**Authors:** Yuichiro Tsunemi, Haruka Nakahigashi

**Affiliations:** ^1^ Department of Dermatology Saitama Medical University Saitama Japan; ^2^ Medical Corporation Shinanokai Shinanozaka Clinic Tokyo Japan; ^3^ Clinical Development Department Kyoto R&D Center Maruho Co., Ltd. Kyoto Japan

**Keywords:** asteatosis, clinical study, finger‐tip unit, moisturizer, volume

## Abstract

Asteatosis is characterized by decreased stratum corneum water content, and the basic treatment is to keep the skin moisturized. Poor application of moisturizers by patients may reduce treatment efficiency, so it is important to continue application as instructed by dermatologists. Application instructions based on the finger‐tip unit are useful for patients, but there is no clear evidence of its efficacy. We investigated the effects of the volume of the moisturizer (Hirudoid^®^ Cream 0.3%) administrated with 1/3 finger‐tip unit and 1 finger‐tip unit equivalent doses per target lower leg of patients with asteatosis (twice daily, 28 days) on the overall dry skin scores, itch numerical rating scale scores, and skin physiological parameters (stratum corneum water content, transepidermal water loss, and skin pH). Sixty patients were randomized with a 1:1 allocation ratio into two groups: the 1/3 finger‐tip unit and 1 finger‐tip unit equivalent dose groups. The results showed that 43.3% of the patients in the 1 finger‐tip unit equivalent dose group, compared with 13.3% in the 1/3 finger‐tip unit equivalent dose group, presented zero overall dry skin scores 1 week later. As the overall dry skin scores improved, the stratum corneum water content also increased. In patients with moderate itching, the itch numerical rating scale scores of the 1 finger‐tip unit equivalent dose group decreased significantly compared with those of the 1/3 finger‐tip unit equivalent dose group. The results suggested that the application of 1 finger‐tip unit equivalent dose of the moisturizer twice daily in clinical practice could induce remission more quickly. With the 1/3 finger‐tip unit equivalent dose, prolonged treatment may be necessary to achieve the desired effect; therefore, application adherence is strictly required. In conclusion, the application of a 1 finger‐tip unit equivalent dose would be quite reasonable in clinical practice.

## 1 INTRODUCTION

Asteatosis, also xeroderma, is caused by decreased water content of the epidermal stratum corneum resulting from a decrease in moisture retention factors (subcorneal intercellular lipids, natural moisturizing factors, sebum, and sweat), and a decrease in the barrier function is observed in some patients.^1^ Factors that cause asteatosis are physiological factors (such as age‐related changes), habits (such as excessive body washing), pathological factors (such as diabetes mellitus, chronic kidney disease, anticancer drug treatment, or dialysis therapy), and environmental factors (such as low humidity and low temperature).^1^, ^2^, ^3^ In patients with asteatosis, the skin becomes white, scaly, and cracked, and is often itchy.^1^, ^2^ Scratching destroys the skin barrier and leads to asteatotic eczema or nummular eczema. In elderly patients, in particular, age‐related reduction of sweating and sebum secretion lead to asteatosis and asteatotic eczema, which are commonly seen in clinical practice. These conditions are accompanied by itching, which causes poor quality of life, including a reduction in daily activities, appetite loss, or sleep disorders.^3^, ^4^ In addition, asteatosis also worsens other skin diseases, such as atopic dermatitis and psoriasis. Thus, it is important to prevent asteatosis.^5^

The cornerstone of treatment for asteatosis is to keep the skin moisturized. Moisturizers are categorized as emollients, such as white petrolatum, and humectants, such as heparinoid preparations and urea preparations. In the case of asteatotic eczema, moisturizers should be continued, with the addition of topical steroids.

Moisturizers are usually applied by patients themselves or by caregivers. Skin care instructions need to be followed during application, especially in terms of volume and frequency of treatment. In dermatology, the finger‐tip unit (FTU) is often used as a guide for the application of topical drugs such as ointment or cream.^6^ One FTU, which is approximately 0.5 g, is the volume of the drug expelled from a tube with a 5‐mm diameter nozzle from the tip to the distal interphalangeal joint of the adult index finger. This volume covers the area of two adult hands, which is approximately 2% of the body surface area. Some reports suggest that application instructions based on the FTU are useful for moisturizers,^7^ but clinical studies have not been conducted to examine whether the 1 FTU equivalent dose is optimal. Due to the lack of reliable evidence, it is difficult for health‐care professionals to instruct an appropriate volume of application of a moisturizer to patients and caregivers, often resulting in misunderstandings or poor adherence.^8^, ^9^ According to an internet survey of 322 Japanese doctors (including dermatologists, pediatricians, and internists) exploring application instructions for prescribed moisturizers in clinical practice, the doctors reported explaining the frequency and timing of application to their patients. Conversely, it was revealed that many doctors frequently do not provide instructions on the volume of application (Internet survey by Macromill Carenet, 30 April 2020, not published).

The Internet survey was conducted on 818 patients or their parents at the same time and revealed that half of the patients who received the instructions about the volume of the moisturizers did not apply a volume equivalent to 1 FTU (Internet survey by Macromill Carenet, 30 April 2020, not published).

In addition, in a study including 62 healthy volunteers, the applied volume that was deemed appropriate without any specific instruction was investigated using topical bases, and there was a large variation (0.2–2.4 FTU equivalent) in volumes applied.^10^ Over half of the volunteers did not apply the volume corresponding to 1 FTU equivalent dose, and the applied volume was instead 0.3–0.4 FTU equivalent dose in the case of half.^10^ These results suggested that failure to understand appropriate volume instructions among patients would result in lower than intended equivalent doses in clinical practice.

Therefore, we investigated the effects of two applied volumes of moisturizer (the 1 FTU equivalent dose, recommended volume, and a 0.3 FTU equivalent dose, estimated volume, assuming no instruction received in clinical practice) on the skin conditions of patients with moderate to severe asteatosis. The investigator’s observations, itch numerical rating scale (NRS) scores, and physiological functions of the skin (stratum corneum water contents [SCWC], transepidermal water loss [TEWL], and skin pH) were observed. The moisturizer selected in this study was a heparinoid preparation, widely used for the treatment of asteatosis in Japan.

## 2 METHODS

### 2.1 Study details

This study was performed in accordance with the Declaration of Helsinki (2013), the Clinical Trials Act (Act No. 16 of 14 April 2017), and the Act on the Protection of Personal Information (Act No. 57 of 30 May 2003). The study protocol was approved by the Certified Review Board of the Medical Corporation Hattori Clinic on 27 January 2020 (CRB No. 3180027). The study was registered in the Japan Registry of Clinical Trials (ID: jRCTs031190200).

This study was performed between 4 February 2020 and 31 March 2020 at the Medical Corporation Shinanokai, Shinanozaka Clinic (Tokyo, Japan). A patient cohort from the Medical Corporation Shinanokai, Shinanozaka Clinic Volunteer Association (Tokyo, Japan) was employed.

Written informed consent was obtained from each patient who wished to participate. Statistical analyses and monitoring were performed by APO PLUS STATION.

### 2.2 Study design

**FIGURE 1:**
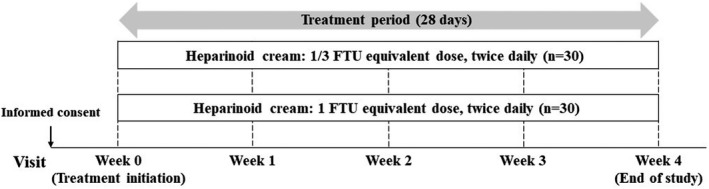
Study design. The patients were subjected to a permuted block randomization process for equal allocation between the two dose groups. If the patients showed eczema after the start of treatment, the rescue treatment (topical administration of steroids) could be added under the direction of the doctor. The study treatment was continued if the overall dry skin score was 0. FTU, finger‐tip unit

This study was performed as a single‐center, randomized, single‐blind (evaluator‐blind), dose‐comparison study with parallel‐dose groups. The patients were randomized with a 1:1 allocation ratio by permuted block method with age (<65 years or ≥65 years) as a stratification factor. The study design is shown in Figure 1.

A fixed volume of the study drug was assigned to each treatment group, whereby approximately 0.5 g (1/3 FTU equivalent dose) was applied in one treatment group, and 1.5 g (1 FTU equivalent dose) was applied in the other group, twice daily for 28 days. The study drug was applied over the entire surface of the target lower leg of each patient in the morning and evening. The patients visited the clinic at week 0, which was the commencement of treatment, week 1, week 2, week 3, and week 4. Their overall dry skin (ODS) scores, itch NRS scores, and physiological functions of the skin were observed. Hirudoid^®^ Cream 0.3% (Maruho) was selected as the moisturizer.

The investigators instructed each patient to apply approximately 0.5 g (1/3 FTU equivalent dose) or 1.5 g (1 FTU equivalent dose) of moisturizer with the measuring spoon supplied by Maruho, and to spread the drug evenly on the target lower leg gently using his/her hand. Moreover, the appropriate volume was ensured in each application by weighing the tube before and after use. The frequency of applications was checked in a patient’s diary with every visit. Instructions to the patients were repeated as required. Patients were also instructed not to apply the moisturizer on the morning of the visit to the clinic. When eczema was observed on the target lower leg, topical steroids could be added as a rescue treatment, at the doctor’s discretion.

### 2.3 Inclusion and exclusion criteria

Patients with asteatosis, with an ODS score of 3 and no sign of eczema in at least one lower leg on the first day of the treatment, and who were at least 20 years of age were enrolled in this study.

The following patients were excluded from the study: (i) patients with a history or presence of serious allergic reactions (shock, anaphylactoid reactions) or hypersensitivity to topical medication; (ii) patients with skin diseases (except asteatosis) on the target lower leg or a bleeding hematological disease (hemophilia, thrombocytopenia, and purpura); (iii) patients who used any topical medicine (including non‐prescription drugs or cosmetics) on the target lower leg within 14 days before treatment initiation; (iv) patients who used an antihistaminic drug within 14 days before treatment initiation; and (v) patients who used oral corticosteroids within 90 days before treatment initiation.

### 2.4 Observations

#### 2.4.1 Overall dry skin scores

**FIGURE 2:**
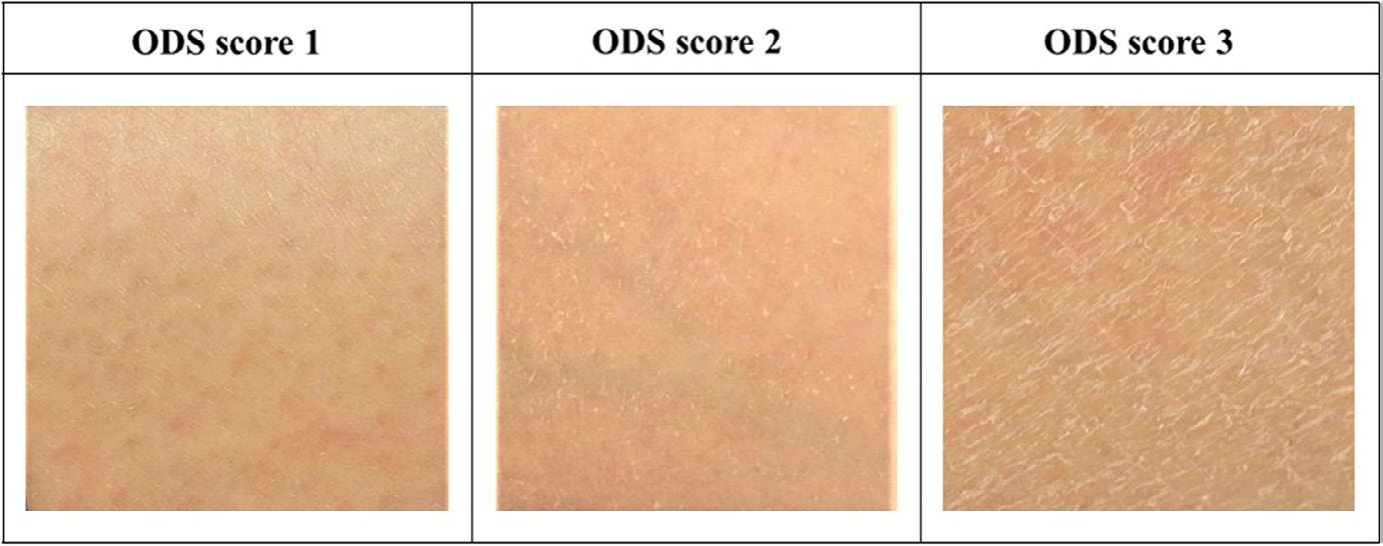
Representative images for each overall dry skin (ODS) score. Each picture shows an ODS score of 0, 1, 2, and 3. Patients with an ODS score of 3 were enrolled in this study; images up to an ODS score of 3 are shown [Color figure can be viewed at wileyonlinelibrary.com]

The dermatologist evaluated the severity of dry skin on the target lower leg using the 5‐point ODS scores: 0, absent; 1, faint scaling, faint roughness, and dull appearance; 2, small scales in combination with a few larger scales, slight roughness, and whitish appearance; 3, small and larger scales uniformly distributed, definite roughness, possible slight redness, and a few superficial cracks; and 4, dominated by large scales, advanced roughness, redness present, eczematous changes, and cracks.^11^ Representative images of the skin for each ODS score are shown in Figure 2.

#### 2.4.2 Itch numerical rating scales scores

The patients evaluated the severity of average itching in the previous 24 h using the NRS scores between 0 (no itching) and 10 (the worst itching they ever experienced).^12^, ^13^

#### 2.4.3 Physiological functions in the skin

The SCWC, TEWL, and skin pH were measured at one site (3 cm × 3 cm in area) of the target lower leg. The SCWC was measured using a Corneometer CM825 device (Courage + Khazaka Electronic). The mean value of five independent measurements at a single site was used for the evaluation. The TEWL was measured using a portable VapoMeter device (Delfin Technologies; Keystone Scientific). The mean value of three measurements at a single site was used for the evaluation. Skin pH was measured using a Skin‐pH‐Meter PH905 (Courage + Khazaka Electronic). The mean value of three measurements at a single site was used for the evaluation.

#### 2.4.4 Adverse events

Information on adverse events (AE) experienced by the patients during the study period was collected for the safety analysis. AE included all unfavorable or unintended symptoms, diseases, and aggravation of present diseases.

### 2.5 Statistical analysis

Adjustment for multiplicity in the test was not performed. All tests were performed as two‐sided tests at a 5% significance level. The two‐sided 95% confidence interval (CI) was also calculated. Results are shown as the mean ± standard deviation (SD).

The ODS and itch NRS scores were compared between the treatment groups at each evaluation time point and compared with those of week 0 in each treatment group. The differences between the treatment groups were compared using the Wilcoxon rank‐sum test, and the differences in each treatment group were compared using the Wilcoxon signed‐rank test.

The SCWC, TEWL, and skin pH were compared in each treatment group with those of week 0 using the paired *t*‐test. The changes in the SCWC and TEWL were compared between the treatment groups at each evaluation time point using the two‐sample *t*‐test.

The correlation between the ODS scores and SCWC was calculated using Pearson’s correlation coefficient.

## 3 RESULTS

### 3.1 Study population, baseline demographics, and clinical characteristics

**FIGURE 3:**
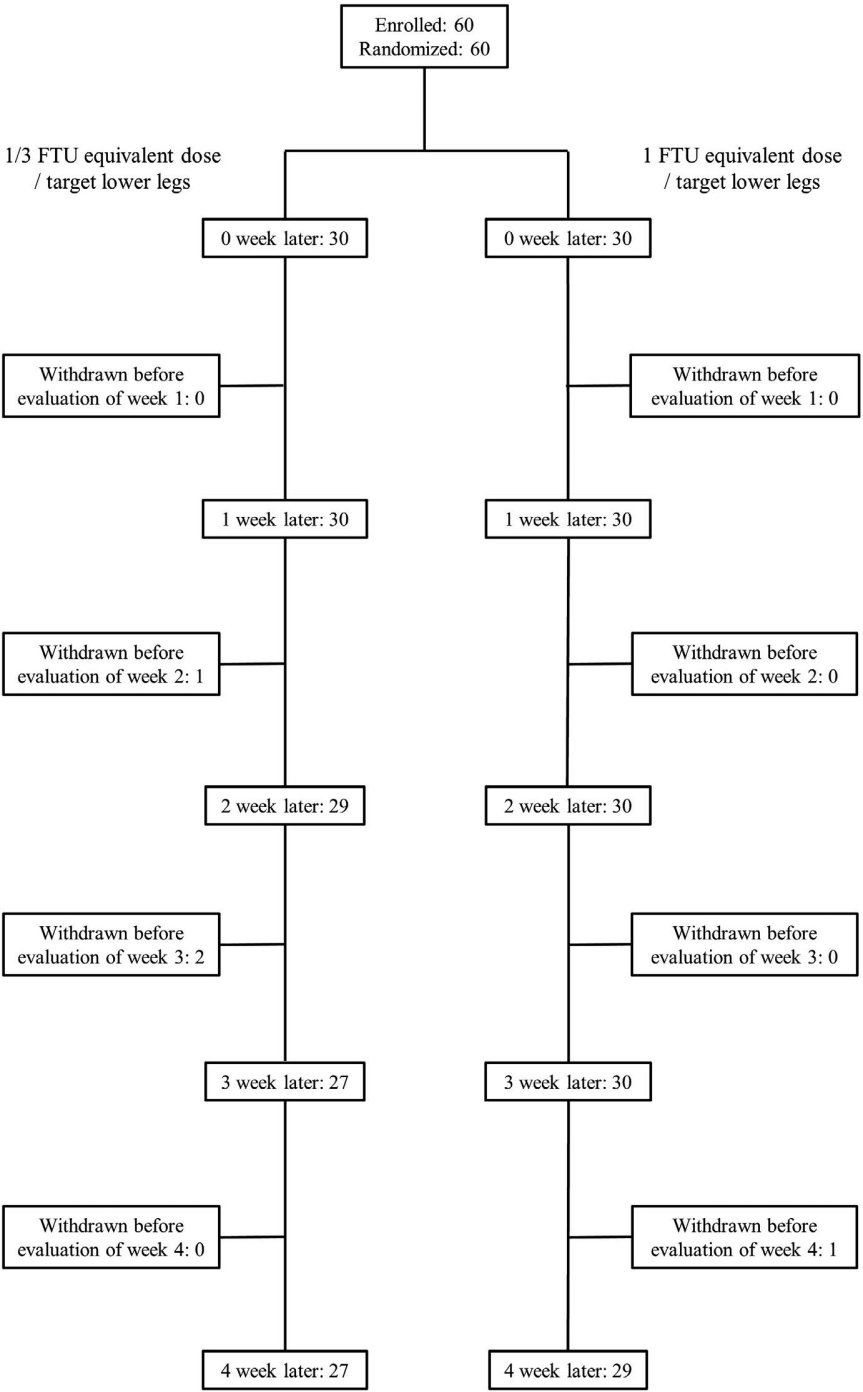
Subject disposition. The investigator decided to discontinue participation in four patients because they did not visit the clinic. FTU, finger‐tip unit

Sixty patients were enrolled in this study (Figure 3). Four patients (three patients in the 1/3 FTU and one patient in the 1 FTU equivalent dose groups) were lost to follow‐up before the evaluation time point at week 4.

Patient characteristics by each dose group are presented in Table 1. There were no differences in the characteristics between the two groups. The mean age ± SD of the patients was 45.5 ± 10.8 and 47.7 ± 9.2 years for the 1/3 FTU and 1 FTU equivalent dose groups, respectively.

**TABLE 1:** Subject characteristics

	1/3 FTU equivalent (n = 30)	1 FTU equivalent (n = 30)	Total (n = 60)
Sex			
Male	11 (36.7%)	12 (40.0%)	23 (38.3%)
Female	19 (63.3%)	18 (60.0%)	37 (61.7%)
Age (years)			
<65	29 (96.7%)	29 (96.7%)	58 (96.7%)
≥65	1 (3.3%)	1 (3.3%)	2 (3.3%)
Lesion site of asteatosis			
Face	2 (6.7%)	11 (36.7%)	13 (21.7%)
Trunk	5 (16.7%)	11 (36.7%)	16 (26.7%)
Femur	9 (30.0%)	10 (33.3%)	19 (31.7%)
Lower legs	30 (100.0%)	30 (100.0%)	60 (100.0%)
Back	5 (16.7%)	6 (20.0%)	11 (18.3%)
Back of the foot	3 (10.0%)	3 (10.0%)	6 (10.0%)
Arm	14 (46.7%)	14 (46.7%)	28 (46.7%)
Others	7 (23.3%)	3 (10.0%)	10 (16.7%)
Target lower leg			
Left lower leg	17 (56.7%)	18 (60.0%)	35 (58.3%)
Right lower leg	13 (43.3%)	12 (40.0%)	25 (41.7%)
Area of target lower leg (cm^2^) (mean ± SD)	840.29 ± 102.9	859.59 ± 113.01	849.94 ± 107.59
Experience in receiving skin care instructions			
No	30 (100.0%)	30 (100.0%)	60 (100.0%)
Yes	0 (0.0%)	0 (0.0%)	0 (0.0%)
Knowledge of FTU			
No	30 (100.0%)	30 (100.0%)	60 (100.0%)
Yes	0 (0.0%)	0 (0.0%)	0 (0.0%)
Complications^a^			
No	19 (63.3%)	22 (73.3%)	41 (68.3%)
Yes	11 (36.7%)	8 (26.7%)	19 (31.7%)
Allergic conjunctivitis	8 (26.7%)	4 (13.3%)	12 (20.0%)
Allergic rhinitis	6 (20.0%)	4 (13.3%)	10 (16.7%)
Diabetes mellitus	1 (3.3%)	0 (0.0%)	1 (1.7%)
Treatment history of asteatosis			
No	26 (86.7%)	27 (90.0%)	53 (88.3%)
Yes	4 (13.3%)	3 (10.0%)	7 (11.7%)

Note

Data are expressed as n (%).

Abbreviations: FTU, finger‐tip unit; SD, standard deviation.

^a^ For details, only typical complications were listed.

Regarding complications, one patient in the 1/3 FTU equivalent dose group had diabetes mellitus, eight and four patients had allergic rhinitis in the 1/3 FTU and 1 FTU equivalent dose groups, respectively, and six and four patients in the 1/3 FTU and 1 FTU equivalent dose groups had allergic conjunctivitis, respectively.

As shown in Table 1, all patients (60/60, 100%) lacked experience in receiving skin care instructions and knowledge of the FTU, and 88.3% of the patients (53/60) had no history of treatment for asteatosis.

### 3.2 Efficacy

#### 3.2.1 Application adherence

The rates of application adherence of the 1/3 FTU and the 1 FTU equivalent dose groups were 99.3% (range, 88.5–100.0%) and 99.7% (range, 96.2–100.0%), respectively. The results of the questionnaire for application instructions showed that 100% and 93.1% of the patients in the 1/3 FTU and 1 FTU equivalent dose groups, respectively, understood the application instructions, and 96.3% and 86.2% of those in the 1/3 FTU and 1 FTU equivalent dose groups, respectively, applied the drug as instructed. The application adherence was high in both groups, with no significant differences. The mean applied volume was 0.561 g (range, 0.48–0.65) for the 1/3 FTU equivalent dose (~0.5 g/target lower leg) group and 1.579 g (range, 1.41–1.77) for the 1 FTU equivalent dose (~1.5 g/target lower leg) group.

#### 3.2.2 Changes in the ODS scores

**FIGURE 4:**
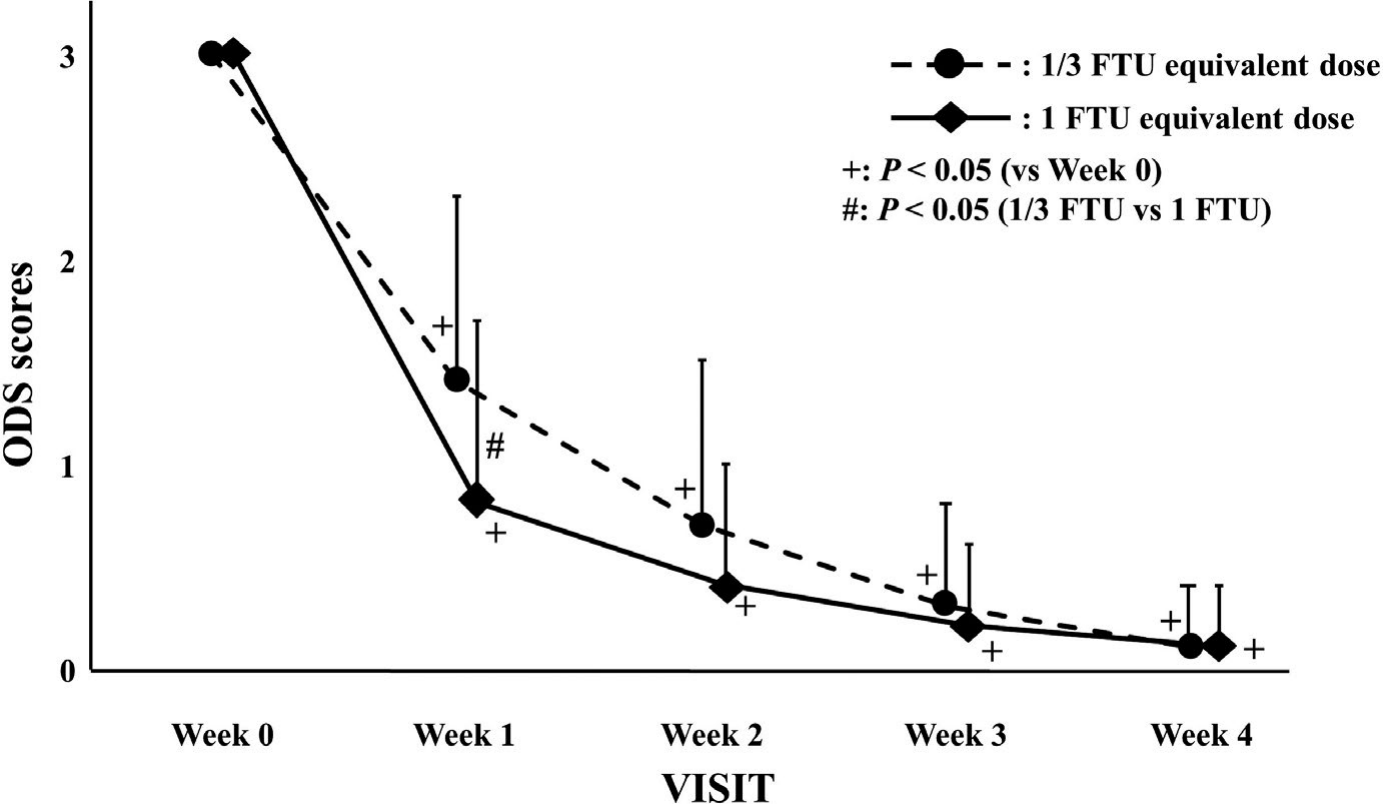
Changes in the overall dry skin (ODS) scores. The circles with a dotted line and rhombi with a solid line indicate the 1/3 finger‐tip unit (FTU) and 1 FTU equivalent dose group, respectively. Error bars represent standard deviations. ^+^Indicates *p *< 0.05 versus week 0 by Wilcoxon signed‐rank test. ^#^Indicates *p *< 0.05 versus between groups by Wilcoxon rank‐sum test

Changes in the ODS scores are shown in Figure 4. The mean ODS scores of the 1/3 FTU and 1 FTU equivalent dose groups were 3.0 and 3.0 in week 0, 1.4 (*p *< 0.001) and 0.8 (*p *< 0.001) in week 1, 0.7 (*p *< 0.001) and 0.4 (*p *< 0.001) in week 2, 0.3 (*p *< 0.001) and 0.2 (*p *< 0.001) in week 3, and 0.1 (*p *< 0.001) and 0.1 (*p *< 0.001) in week 4 (*p* vs. week 0), respectively. The mean ODS scores in both groups decreased significantly compared with those at week 0. No patient had to receive rescue treatment.

A total of 43.3% of the patients in the 1 FTU equivalent dose group showed zero ODS scores in week 1. In contrast, in the 1/3 FTU equivalent dose group, a score of 0 was observed in only 13.3% of the patients. At week 1, the ODS scores of the 1 FTU equivalent dose group were significantly lower than those of the 1/3 FTU equivalent dose group (*p* = 0.015).

#### 3.2.3 Changes in itch NRS scores

**FIGURE 5:**
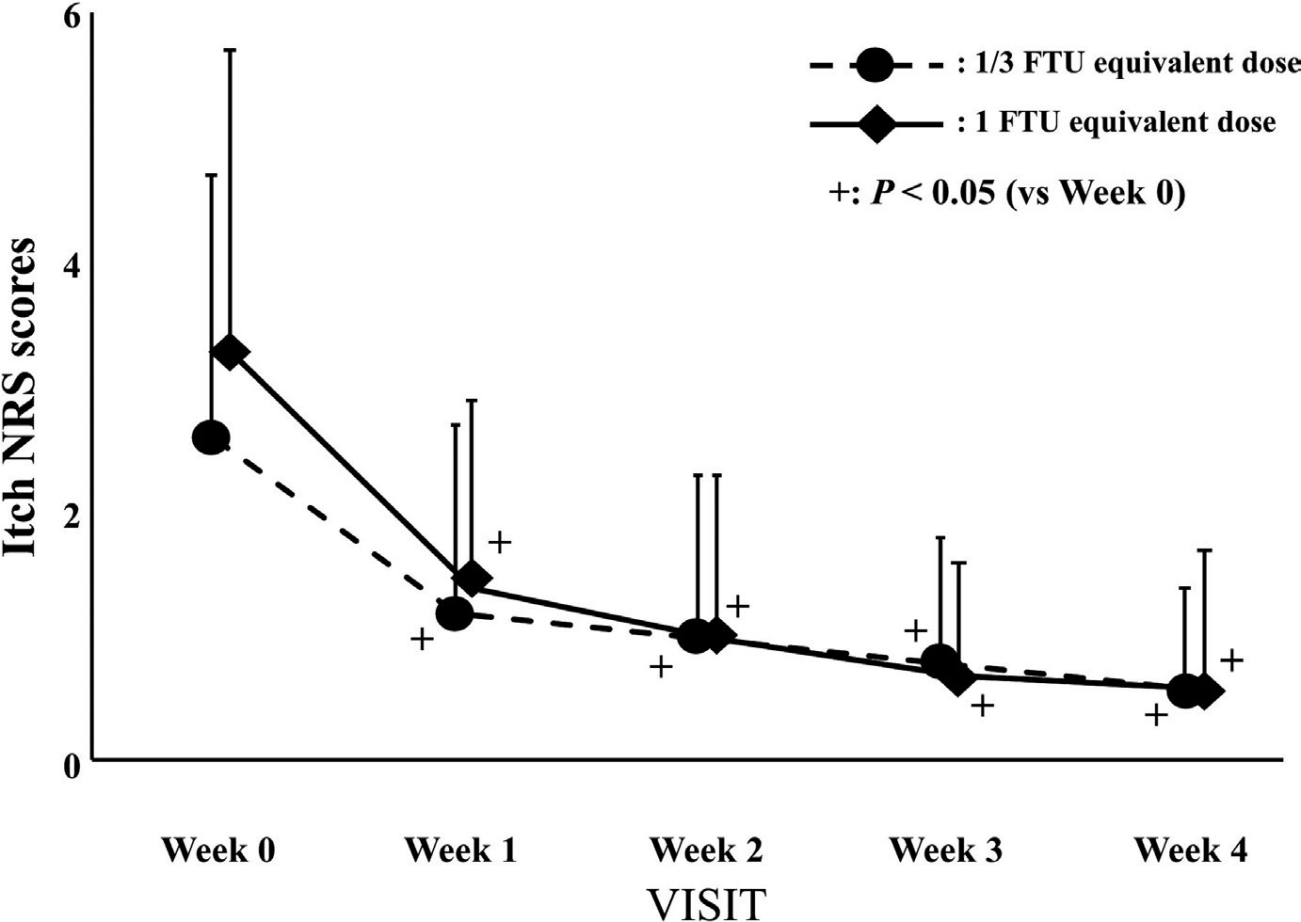
Changes in the itch numerical rating scale (NRS) scores. The circles with a dotted line and rhombi with a solid line indicate the 1/3 finger‐tip unit (FTU) and 1 FTU equivalent dose group, respectively. Error bars represent standard deviations. ^+^Indicates *p *< 0.05 versus week 0 by Wilcoxon signed‐rank test

Changes in the itch NRS scores are shown in Figure 5. Almost all patients (24/30 in the 1/3 FTU and 28/30 in the 1 FTU equivalent dose group, respectively) experienced itching (NRS scores 1–8) in week 0. The mean itch NRS scores of the 1/3 FTU and 1 FTU equivalent dose groups were 2.6 and 3.3 in week 0, 1.2 (*p *< 0.001) and 1.4 (*p *< 0.001) in week 1, 1.0 (*p *< 0.001) and 1.0 (*p *< 0.001) in week 2, 0.8 (*p *< 0.001) and 0.7 (*p *< 0.001) in week 3, and 0.6 (*p *< 0.001) and 0.6 (*p *< 0.001) in week 4 (*p* vs. week 0), respectively. The mean itch NRS scores of both groups decreased significantly over time compared with those at week 0. The mean itch NRS score of the 1 FTU equivalent dose group (3.3) was somewhat higher than that of the 1/3 FTU equivalent dose group (2.6) in week 0, but it decreased to comparable scores (1.4 and 1.2, respectively) in week 1, followed by similar profiles in weeks 2, 3, and 4.

**FIGURE 6:**
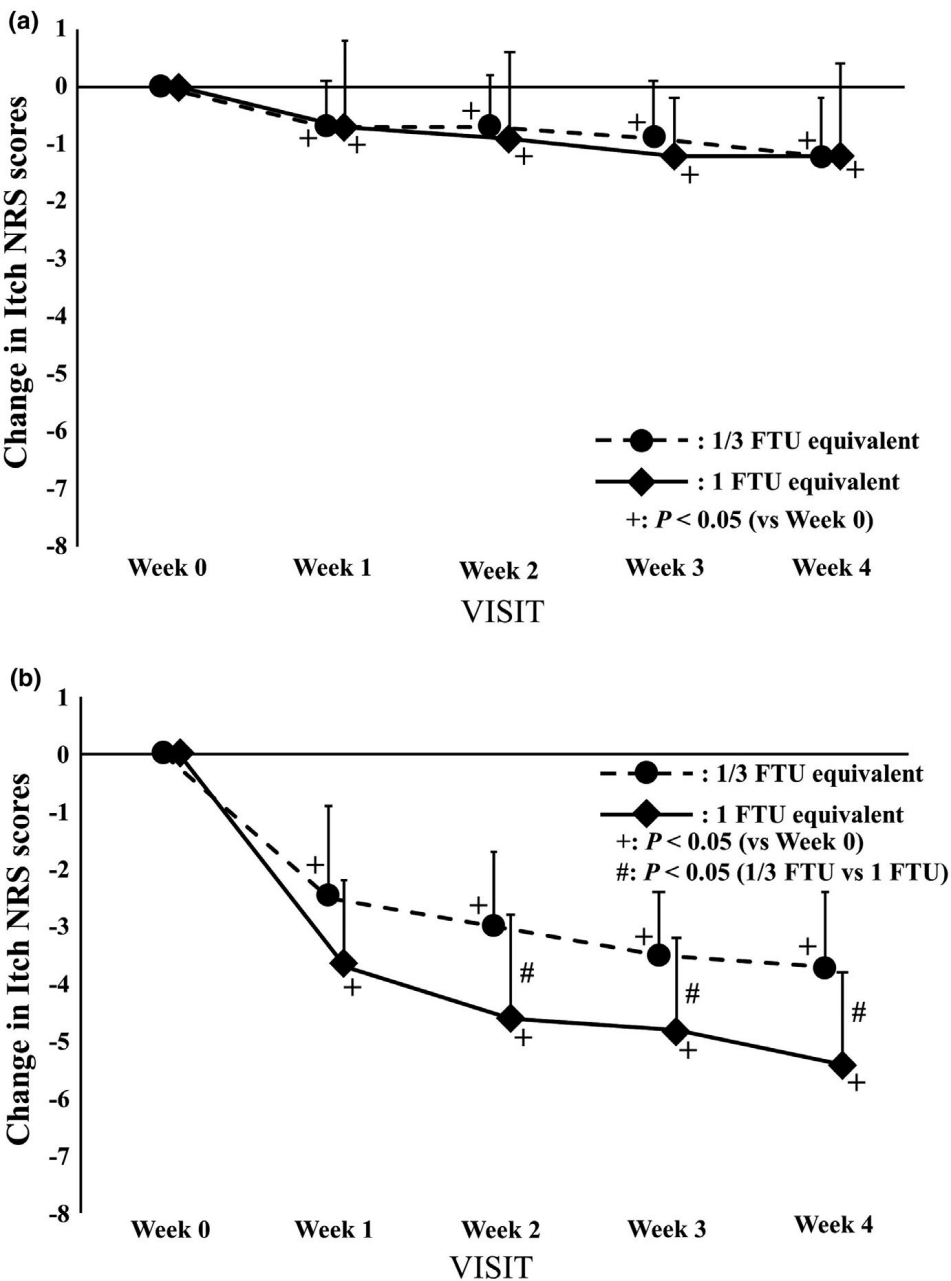
Changes in the itch numerical rating scale (NRS) scores. The data at each evaluation point are shown as the amount of change with respect to the value at week 0. The circles with a dotted line and rhombi with a solid line indicate the 1/3 finger‐tip unit (FTU) and 1 FTU equivalent dose group, respectively. Error bars represent standard deviations. ^+^Indicates *p *< 0.05 versus week 0 by Wilcoxon signed‐rank test. ^#^Indicates *p *< 0.05 versus between groups by Wilcoxon‐rank sum test. (a) Patients with mild itch: ≤3 (1/3 FTU equivalent dose group: n = 17–19; 1 FTU equivalent dose group: n = 18–19). (b) Patients with moderate itch: ≥4 (1/3 FTU equivalent dose group: n = 10–11; 1 FTU equivalent dose group: n = 11)

We classified and analyzed cases according to the degree of mild and moderate itch, regarding the itch NRS scores of 3 or less as mild and 4 or more as moderate.^12^, ^13^ In the patients with mild itch, no difference in the changes in itch NRS scores was observed between the two groups (Figure 6a). Conversely, in the patients with moderate itch, the changes in itch NRS scores of the 1 FTU equivalent dose group were significantly lower than those of the 1/3 FTU equivalent dose group (*p *< 0.05, Figure 6b).

### 3.3 Physiological functions in the skin

The physiological parameters are summarized in Table 2. No differences in baseline characteristics for each parameter were observed between the groups.

**TABLE 2:** Changes over time of SCWC, TEWL, and skin pH

	Week	1/3 FTU equivalent dose group	*p*‐value Intragroup*	1 FTU equivalent dose group	*p*‐value Intragroup*
SCWC (AU)	Week 0	17.86 ± 7.51 (30)	–	16.97 ± 7.42 (30)	–
	Week 1	32.10 ± 10.24 (30)	<0.001	36.70 ± 7.25 (30)	<0.001
	Week 2	34.11 ± 7.54 (29)	<0.001	34.11 ± 6.63 (30)	<0.001
	Week 3	35.05 ± 6.82 (27)	<0.001	33.89 ± 5.81 (30)	<0.001
	Week 4	36.30 ± 5.22 (27)	<0.001	34.09 ± 5.75 (29)	<0.001
TEWL (g/m^2^/h)	Week 0	8.15 ± 1.65 (30)	–	8.08 ± 1.70 (30)	–
	Week 1	7.84 ± 1.72 (30)	0.181	8.05 ± 2.04 (30)	0.884
	Week 2	7.09 ± 1.69 (29)	<0.001	7.27 ± 1.66 (30)	<0.001
	Week 3	6.54 ± 1.50 (27)	<0.001	6.58 ± 1.75 (30)	<0.001
	Week 4	6.55 ± 1.55 (27)	<0.001	6.80 ± 1.90 (29)	<0.001
Skin pH	Week 0	5.137 ± 1.362 (30)	–	5.434 ± 1.933 (30)	–
	Week 1	4.972 ± 0.806 (30)	0.513	5.381 ± 0.728 (30)	0.895
	Week 2	5.085 ± 1.867 (29)	0.838	4.682 ± 0.980 (30)	0.071
	Week 3	4.680 ± 0.690 (27)	0.065	4.975 ± 0.796 (30)	0.207
	Week 4	4.408 ± 0.913 (27)	0.037	4.734 ± 0.731 (29)	0.081

Note

Data are expressed as means ± SD (*n*). *p‐value for intragroup comparison between Week 0 and each evaluation point.

Abbreviations: FTU, finger‐tip unit; SCWC, stratum corneum water content; SD, standard deviation; TEWL, transepidermal water loss.

Significant differences in the measured values of males and females were observed in the TEWL (males: 9.06 ± 1.33; females: 7.52 ± 1.59; *p *< 0.001) and skin pH (males: 4.636 ± 1.303; females: 5.690 ± 1.751; *p *< 0.016), but no differences in the SCWC were observed.

**FIGURE 7:**
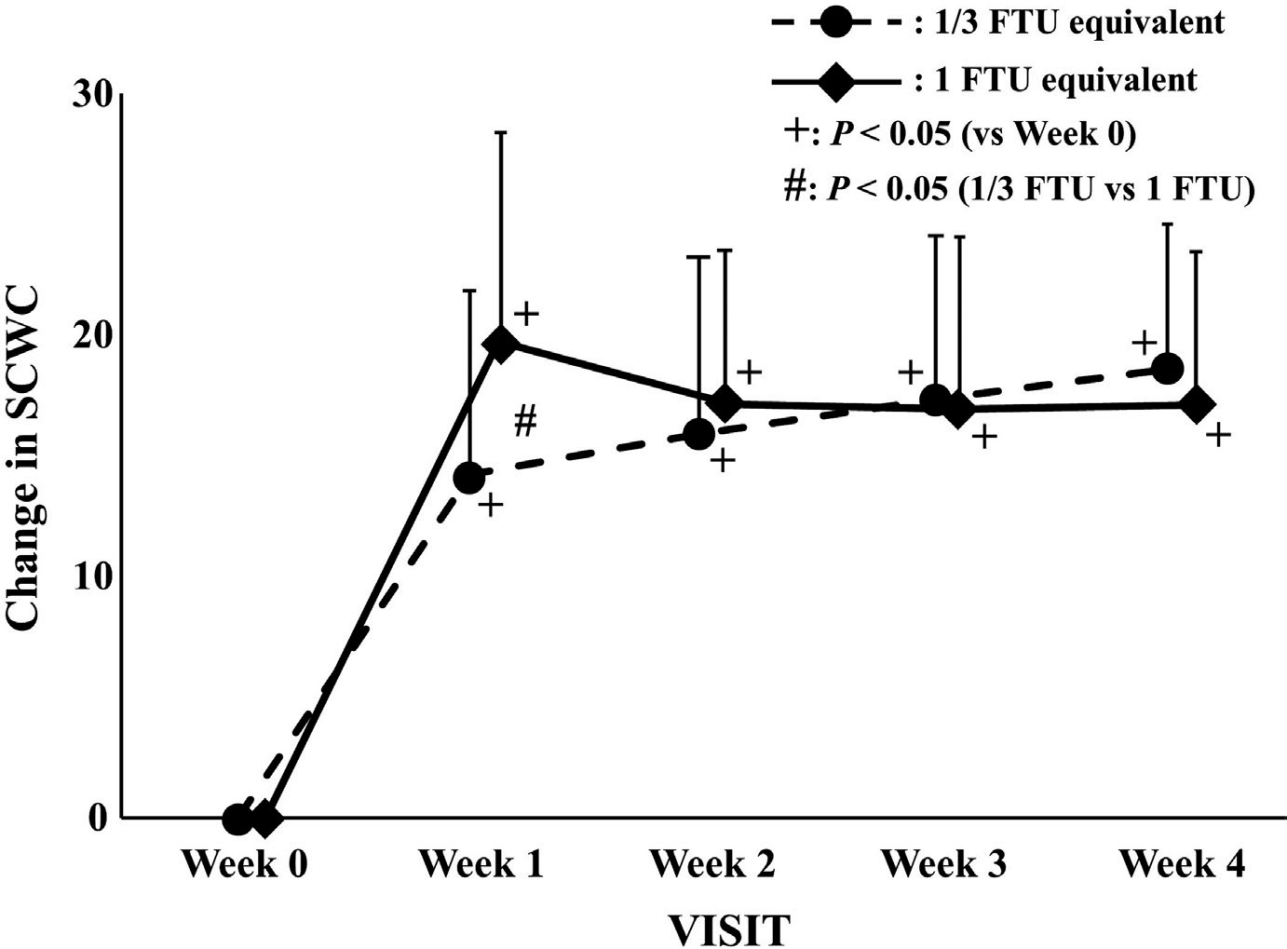
Changes in the stratum corneum water contents (SCWC). The data at each evaluation point are shown as the amount of change with respect to the value at week 0. The circles with a dotted line and rhombi with a solid line indicate the 1/3 finger‐tip unit (FTU) and 1 FTU equivalent dose group, respectively. Error bars represent standard deviations. ^+^Indicates *p *< 0.05 versus week 0 by paired *t*‐test. ^#^Indicates *p *< 0.05 versus between groups by two‐sample *t*‐test

The changes in the SCWC (1/3 FTU equivalent dose vs. 1 FTU equivalent dose) were 14.42 versus 19.73 in week 1 (*p* = 0.011), 15.94 versus 17.14 in week 2 (*p* = 0.502), 17.36 versus 16.92 in week 3 (*p* = 0.813), and 18.61 versus 17.11 in week 4 (*p* = 0.366), respectively (Figure 7). The changes in the SCWC of the 1 FTU equivalent dose group were significantly larger in week 1 compared with those of the 1/3 FTU equivalent dose group.

**FIGURE 8:**
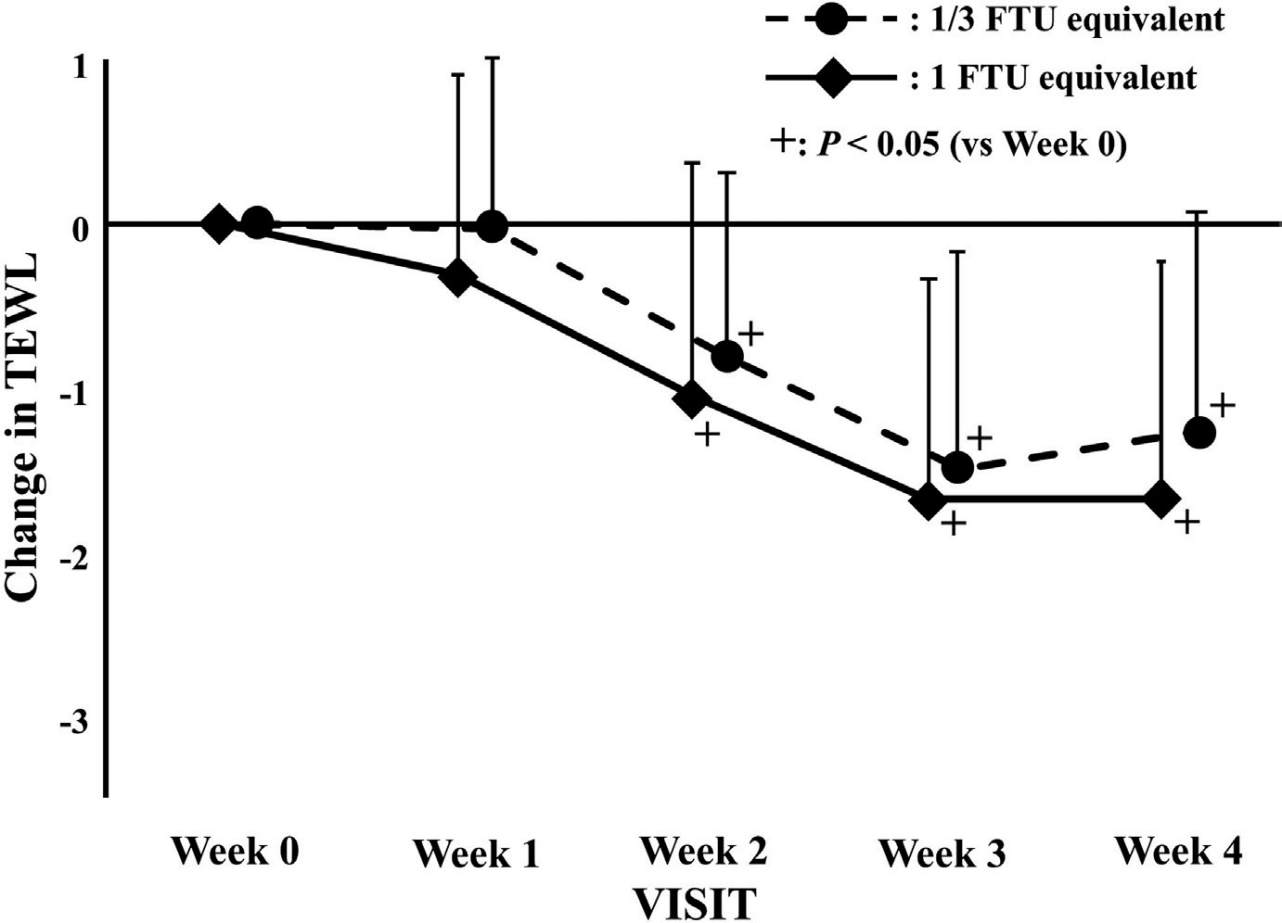
Changes in transepidermal water loss (TEWL). The data at each evaluation point are shown as the degree of change with respect to the value at week 0. The circles with a dotted line and rhombi with a solid line indicate the 1/3 finger‐tip unit (FTU) and 1 FTU equivalent dose group, respectively. Error bars represent standard deviations. ^+^Indicates *p *< 0.05 versus week 0 by paired *t*‐test

The TEWL decreased significantly in weeks 2, 3, and 4 in both groups compared with that in week 0 (*p *< 0.001) but reached a plateau in weeks 3 and 4 in both groups (Table 2). There was no difference between the changes in either group (Figure 8).

The skin pH was slightly acidic at week 4 compared with week 0 in both groups (Table 2). There was no significant difference between the groups.

### 3.4 Relationship between skin symptoms and physiological functions

The ODS scores decreased significantly, and the SCWC increased significantly. A correlation was demonstrated between the changes in the ODS scores and the SCWC in week 1 (correlation coefficient: −0.4263, *p* = 0.001).

### 3.5 Safety

Three AE of itching were observed in three patients in the 1 FTU equivalent dose group, and none of these were serious events. All patients continued the study and recovered without any medical treatment.

## 4 DISCUSSION

Asteatosis is a disease resulting from a decrease in the water content of the epidermal stratum corneum and may induce inflammatory cascades that trigger itch.^1^, ^14^ Patients with asteatosis often experience symptoms of itching.^1^, ^2^ Scratching the skin damages the barrier function and results in the worsening of symptoms. Therefore, early moisturizing care of the affected region would be very important in patients with asteatosis to prevent the progression of symptoms.^1^, ^14^ Unlike oral medications, the treatment effects of topical medication greatly depend on the adherence and understanding of each patient, which tend to decline in clinical practice.^15^, ^16^, ^17^

Moisturizers are known to exert a more beneficial effect when applied twice daily rather than once daily.^18^, ^19^ “Clinical practice guidelines for the management of atopic dermatitis 2018” in Japan state that moisturizers should be applied twice daily.^20^ As for the volume of moisturizer to be applied, some reports recommended applying a 1 FTU equivalent dose,^7^ but the recommended volume has not been supported by clinical studies. Therefore, we focused our study on the evaluation of outcomes based on the applied volume of moisturizers.

We investigated whether the treatment outcomes with the application of moisturizer twice daily were different between the 1 FTU equivalent dose (~ 1.5 g/target lower leg) and 1/3 FTU equivalent dose (~0.5 g/target lower leg) in patients with moderate to severe asteatosis. The 1/3 FTU equivalent dose was selected as “a usual dose in clinical practice” because it was considered appropriate by patients who had not received any dosage instructions from their treating clinician.^10^

The results after twice daily treatment with the moisturizer showed that the dry symptoms of the skin in the 1 FTU equivalent dose group improved earlier than in the 1/3 FTU equivalent dose group, and 43.3% of the 1 FTU equivalent dose group reported zero ODS scores at week 1. Furthermore, in the patients with moderate itching, the changes in itch NRS scores in the 1 FTU equivalent dose group decreased significantly compared with those in the 1/3 FTU equivalent dose group at weeks 2, 3, and 4. Despite the difference in the initial scores (1/3 FTU equivalent dose: 4.9 ± 1.2; 1 FTU equivalent dose: 6.0 ± 1.3; *p* = 0.060), the scores of both groups decreased to the same level (1/3 FTU equivalent dose: 1.2 ± 0.8; 1 FTU equivalent dose: 0.6 ± 0.9; *p* = 0.151). These results indicate that topical instructions based on the 1 FTU equivalent dose (the traditionally recommended volume) are more effective at improving symptoms of asteatosis.

In this study, the investigators verified whether the patients were able to apply the moisturizer as instructed, and if necessary, they were instructed again, so high adherence was maintained. If such a high level of adherence is maintained, sufficient treatment effects might also be obtained even in the 1/3 FTU equivalent dose group because the ODS scores of the 1/3 FTU equivalent dose group achieved comparable improvements to those of the 1 FTU equivalent dose group until week 4.

However, adherence tends to be markedly enhanced in clinical trials and research settings. Many factors complicate treatment with topical medications; hence, it is difficult to maintain adherence in clinical practice. Some reports have shown that adherence to topical medication in patients with atopic dermatitis was high (93%) 3 days after the dermatologist’s careful examination, but it markedly decreased to 32% after eight weeks.^21^ In addition, one of the reasons why the adherence of patients decreases is that patients feel anxious or dissatisfied with the topical medication due to ineffectiveness.^8^ Based on the above difficulties in maintaining adherence in clinical practice, the application of 1 FTU equivalent of the moisturizer is desirable.

Examinations of artificial dry skin of healthy volunteers suggested that the SCWC increases depending on the applied volume.^20^, ^21^ In this study, however, the treatment effect of a 2 FTU equivalent dose was not considered as this is considered to be an excessive amount for application, and it will not remain on the skin because a portion of the coating drug attaches to clothes in daily life.

The improvement in skin symptoms was also supported by an increase in SCWC. Compared with the quick improvement of the ODS scores and the SCWC, the TEWL began to decrease at week 2 or later, suggesting that the improvement of the skin barrier function requires more time. Furthermore, we found the pH of the skin to be slightly acidic at week 4. Of note, the restoration of the skin barrier function has been reported to be hampered in alkaline conditions.^22^, ^23^, ^24^, ^25^

This study had some limitations. First, we used only a heparinoid preparation (Hirudoid^®^ Cream 0.3%) as a moisturizer, and the effects of other moisturizers need to be investigated further. Second, the number of elderly patients was small.

In conclusion, we investigated the effects of two different applied volumes (1/3 FTU and 1 FTU equivalent doses) of the moisturizer. Among patients assigned to the 1 FTU equivalent dose group, the ODS scores improved significantly in week 1, and more patients had zero scores compared with those assigned to the 1/3 FTU equivalent dose group. The results suggest that the application of 1 FTU equivalent dose of the moisturizer twice a day in clinical practice could induce remission more quickly. With 1/3 FTU equivalent dose, prolonged treatment may be necessary until the desired treatment effect is achieved, and high adherence to the treatment is strictly required. Therefore, the 1 FTU equivalent dose would be quite reasonable for application instruction in clinical practice.

## CONFLICT OF INTEREST

This study was funded by Maruho. Y.T. received consultant and speaker fees from Maruho, and H.N. is an employee of Maruho.

## References

[1] Kikuchi K , Igarashi A , Katoh N , Ikoma A , Kanakubo A , Terui T . Review of asteatosis (dry skin) –definition and treatment consideration of asteatosis–. Jpn J Dermatol. 2019;129:2763–70. (In Japanese).

[2] Tagami H . Xeroderma and xerodermatic dermatitis. MB Derma. 2002;57:2–8. (In Japanese).

[3] Yaguchi H . Home treatment for eczema/dermatitis. MB Derma. 2010;161:33–8. (In Japanese).

[4] Kobayashi Y . Skin care for the elderly–From the perspective of the certified nurse in wound, ostomy, and continence nursing–. Geriat Med. 2012;50:855–9. (In Japanese).

[5] Tsunemi Y , Kawashima M . Correspondence to the skin diseases in the elderly: mycosis and eczema/dermatitis. Kango Kenkyu. 2017;50:370–9. (In Japanese).

[6] Long CC , Finlay AY . The finger‐tip unit–a new practical measure. Clin Exp Dermatol. 1991;16:444–7. 1806320 10.1111/j.1365-2230.1991.tb01232.x

[7] Miyachi Y . The application quantity quantification tool: how to use the FTU and precautions. J Visual Dermatol. 2017;16:462–4. (In Japanese).

[8] Nakahara T . Treatment adherence for atopic dermatitis. Allergy Pract. 2016;36:1132–6. (In Japanese).

[9] Pouplard C , Gourraud P‐A , Meyer N , Livideanu CB , Lahfa M , Mazereeuw‐Hautier J , et al. Are we giving patients enough information on how to use topical treatments? Analysis of 767 prescriptions in psoriasis. Br J Dermatol. 2011;165:1332–6. 21711325 10.1111/j.1365-2133.2011.10480.x

[10] Nakahigashi H , Numazaki T , Nakamura H , Yoshioka D , Ashizuka Y , Yoshimune R , et al. Clinical research on the effects of differences in properties of external preparations for skin on application amount: An exploratory investigation of the relationship between the base usability and application amount by questionnaire in adult subjects. Yakugaku Zasshi. 2019;139:1313–25. (In Japanese). 31231102 10.1248/yakushi.19-00091

[11] Serup J . EEMCO guidance for the assessment of dry skin (xerosis) and ichthyosis: clinical scoring systems. Skin Res Technol. 1995;1:109–14. 27328437 10.1111/j.1600-0846.1995.tb00029.x

[12] Phan N , Blome C , Fritz F , Gerss J , Reich A , Ebata T , et al. Assessment of pruritus intensity: prospective study on validity and reliability of the visual analogue scale, numerical rating scale and verbal rating scale in 471 patients with chronic pruritus. Acta Derm Venereol. 2012;92:502–7. 22170091 10.2340/00015555-1246

[13] Ständer S , Augustin M , Reich A , Blome C , Ebata T , Phan N , et al. Pruritus assessment in clinical trials: consensus recommendations from the international forum for the study of itch (IFSI) special interest group scoring itch in clinical trials. Acta Derm Venereol. 2013;93:509–14. 23624777 10.2340/00015555-1620

[14] Tsunemi Y , editor. Textbook of topical medication for everyone. Tokyo: Nankodo; 2019. (In Japanese).

[15] Torrelo A , Ortiz J , Alomar A , Ros S , Pedrosa E , Cuervo J . Health‐related quality of life, patient satisfaction, and adherence to treatment in patients with moderate or severe atopic dermatitis on maintenance therapy: the CONDA‐SAT study. Actas Dermosifiliogr. 2013;104:409–17. 23665434 10.1016/j.adengl.2013.04.004

[16] Conlon NP , Edgar JDM . Adherence to best practice guidelines in chronic spontaneous urticaria (CSU) improves patient outcome. Eur J Dermatol. 2014;24:385–6. 24682625 10.1684/ejd.2014.2323

[17] Snyder S , Crandell I , Davis SA , Feldman SR . Medical adherence to acne therapy: a systematic review. Am J Clin Dermatol. 2014;15:87–94. 24481999 10.1007/s40257-014-0063-y

[18] Nakamura M , Uemura K , Nemoto O , Miyachi Y . Evaluation of an optimal method for topical application of moisturizer. Skin Res. 2006;5:311–6. (In Japanese).

[19] Ohtani M , Ohtani M , Nozawa A , Matsumoto M , Yamamura Y , Komoda M , et al. A study of the influence of the volume and frequency of application on the efficacy of moisturizers. Jpn J Dermatol. 2012;122:39–43. (In Japanese).

[20] Katoh N , Ohya Y , Ikeda M , Ebihara T , Katayama I , Saeki H , et al. Clinical practice guidelines for the management of atopic dermatitis 2018. J Dermatol. 2019;46:1053–101. 31599013 10.1111/1346-8138.15090

[21] Shah A , Yentzer BA , Feldman SR . Timing of return office visit affects adherence to topical treatment in patients with atopic dermatitis: an analysis of 5 studies. Cutis. 2013;91:105–7. 23513560

[22] Hachem J‐P , Crumrine D , Fluhr J , Brown BE , Feingold KR , Elias PM , et al. pH directly regulates epidermal permeability barrier homeostasis, and stratum corneum integrity/cohesion. J Invest Dermatol. 2003;121:345–53. 12880427 10.1046/j.1523-1747.2003.12365.x

[23] Ali SM , Yosipovitch G . Skin pH: from basic science to basic skin care. Acta Derm Venereol. 2013;93:261–7. 23322028 10.2340/00015555-1531

[24] Eyerich S , Eyerich K , Traidl‐Hoffmann C , Biedermann T . Cutaneous barriers and skin immunity: differentiating a connected network. Trends Immunol. 2018;39:315–27. 29551468 10.1016/j.it.2018.02.004

[25] Hülpüsch C , Tremmel K , Hammel G , et al. Skin pH‐dependent *Staphylococcus aureus* abundance as predictor for increasing atopic dermatitis severity. Allergy. 2020;75:2888–98. 32562575 10.1111/all.14461

